# Propofol Alleviates Intractable Migraine Headache: A Case Report

**DOI:** 10.5812/aapm.7358

**Published:** 2012-09-13

**Authors:** Masood Mohseni, Farzad Fatehi

**Affiliations:** 1 Department of Anesthesiology, Rasoul Akram Medical Center, Iran University of Medical Sciences (IUMS), Tehran, IR Iran; 2 Department of Neurology, Tehran University of Medical Sciences (TUMS), Tehran, IR Iran

**Keywords:** Pain, Migraine Disorders, Propofol, Anesthetics

## Abstract

**Abstract:**

Several medications have been proposed as the first line drugs in the treatment of migraine attack. However, the low efficacy, potential complications of medications and the intolerance of some patients for oral route due to nausea and vomiting establish a difficult situation in some migraine patients. This report describes a dramatic pain relief with 60 mg of intravenous propofol in a patient with migraine attack refractory to treatment with metoclopramide, promethazine, dexamethasone and meperidine. Pain relief in this patient besides earlier case reports suggests that subanesthetic doses of propofol may be an alternative to other treatment modalities for acute migraine.

## 1. Introduction

Migraine headache is a disabling clinical entity and a frequent cause of attendance to the emergency department. Several medications including intravenous non-steroidal anti-inflammatory drugs, paracetamol, triptans, Phenothiazines, new atypical antipsychotics, metoclopramide and opioids have been proposed as the first line drugs in the treatment of migraine attack (1-6). However, the low efficacy, potential complications of medications and the intolerance of some patients for oral route due to nausea and vomiting, establish a difficult situation in some migraine patients.

Anecdotal evidence suggests that propofol may be effective at alleviating migraine headache. Nevertheless, the current knowledge is founded on a limited number of case reports with discriminated situations. A greater body of evidence with more varied case reports is required before suggesting the routine clinical application of this treatment or even conducting clinical trials. This report describes a dramatic pain relief with propofol in a patient with migraine attack unresponsive to metoclopramide, promethazine and meperidine.

## 2. Case Presentation

A 29-year-old woman was admitted to the emergency department for severe migraine headache accompanied by nausea and vomiting from four hours before admission. Her neurological exam was normal and the quality and pattern of headache was similar to her prior migraine attacks. She expressed the quality of her headache as a sharp, pulsating pain located in the area of the left temple and forehead. The patient had received dexamethasone 8 mg and meperidine 25 mg 2 hours before admission in a day clinic but the pain was not subsided. On admission, she received metoclopramide 20 mg diluted in 500 mL of normal saline plus meperidine 25 mg and promethazine 50 mg. About 20 minutes later the patients showed jerky movements in her hands while fully awake and still with complaint of severe headache. The jerky movements were attributed to the extrapyramidal complications of metoclopramide. One mL of Intravenous lidocaine 2% and 6 mL of propofol 1% was administered for the patient. The patient fell asleep after a few seconds for more than six hours. After waking up, the patient was completely pain free without any nausea or vomiting. She stated that her migraine attacks always had lasted more than the recent headache and she was now completely pain free and seldom had experienced a state of well-being like that. On follow-up call visit 72 hours after discharge, she was well without any headache.

On history taking, our patient was on maintenance doses of topiramate and amitriptyline for her frequent migraine attacks. In spite of preventive treatments, she suffered from weekly attacks of migraine headache. Once started, her headaches were not responsive to oral medications including acetaminophen codeine and ibuprofen, and often required intravenous opioids for pain relief and ordinarily, the headaches lasted over 36 hours.

## 3. Discussion

The dramatic pain relief in our patient adds to the body of evidence on the therapeutic effects of propofol in migraine headache. The first and most important report of propofol administration for headache was on a mixture of 77 patients suffering from migraine or non-migrainous headache refractory to the usual methods of abortive treatment. In this report, subanesthetic doses of propofol effectively relieved the patient’s headache. 63 of 77 patients in this study reported complete abolition of their headache and only three of the treated patients reported a return of the headache on the day following treatment (7). Although reported to be effective in a diverse mixture of headaches, this report cannot identify those patients who most benefit from propofol administration.

Two major mechanisms have been proposed for the development of migraine: cortical spreading depression and cortical hyperexcitability or central sensitization. CSD is triggered when enhanced cortical activity coincides with other triggering factors. CSD induces the release of a variety of vasoactive and inflammatory substances which makes a condition known as sterile neurogenic inflammation (8). This phenomenon could activate and/or sensitize the meningeal trigeminal afferents and induce migraine or its aura. Recently it has proposed that propofol can suppress CSD, which makes it a potential medication for migraine patients (9). Noteworthy, CSD is believed to be the neurogenic correlate of migraine aura while our patient did not experience any prodromal symptoms. This suggests that mechanisms other than CSD suppression may be involved in the therapeutic effects of propofol.

Another suggested theory for the development of migraine is central sensitization. Central sensitization points to the pain response to non-painful stimuli known as allodynia or decreased pain threshold, clinically presented as hyperalgesia (10). Propofol as an anesthetic with multiple effects on the neurotransmitters in the central nervous system may diminish the central sensitization. Noteworthy, the observed therapeutic effects were demonstrated in the subanesthetic doses. The mechanism of pain reduction of propofol may also be attributed to the GABA agonistic effects as well as its cerebral vasoconstriction effects.

The headache in our patients was not subsided with the administration of dexamethasone, promethazine, metoclopramide and even meperidine. These drugs are routinely used in the emergency departments for the treatment of migraine attacks and several studies support their therapeutic efficacy (1-7). Pain relief in our patient along with earlier case reports suggests that subanesthetic doses of propofol may be an alternative to other treatment modalities for acute migraine. Regarding the potential life-threatening complications of propofol such as apnea, significant hypotension or a wide range of allergic reactions, it should be used cautiously under the discretion of a physician who is familiar with this anesthetic. Further research is required to clarify the dosing regimen as well as target patients who most benefit from this treatment modality.
